# Follicular thyroid carcinoma: differences in clinical relevance between minimally invasive and widely invasive tumors

**DOI:** 10.1186/s12957-015-0612-8

**Published:** 2015-06-04

**Authors:** Mauro Podda, Alessandra Saba, Federica Porru, Isabella Reccia, Adolfo Pisanu

**Affiliations:** Department of Surgery, University of Cagliari, Azienda Ospedaliero-Universitaria, Presidio Policlinico di Monserrato, Blocco G, SS 554 km 4, 500 - 09042 Monserrato, Cagliari Italy

**Keywords:** Follicular thyroid carcinoma, Minimally invasive carcinoma, Widely invasive carcinoma, Lymph node metastasis, Recurrence, Prognosis

## Abstract

**Background:**

Evidence on the biological behavior and clinical courses of minimally invasive and widely invasive follicular thyroid carcinoma (MI-FTC, WI-FTC) is still debatable. The current study was conducted to identify differences between MI and WI tumors and those prognostic parameters influencing late outcome such as local recurrence and survival.

**Methods:**

From January 1998 to October 2013, 71 patients were operated on in our department because of a FTC. A retrospective cohort study was carried out to compare 42 MI-FTC and 29 WI-FTC. The comparison involved evaluation of patient characteristics, tumor characteristics, tumor staging, and risk assessment.

**Results:**

A diameter greater than 4.0 cm, the presence of vascular invasion, the TNM stage III–IVA, and the high risk at AMES system risk stratification were independent factors significantly related to the presence of a WI-FTC. The only independent predictor of recurrence and disease-free survival at 10-year follow-up was a tumor size greater than 4.0 cm.

**Conclusions:**

More attention must be paid in the postoperative tumor re-staging of those patients with tumor size larger than 4.0 cm, which was the only parameter predicting recurrence and influencing disease-free survival. Nevertheless, definitive recommendations cannot be made without a longer follow-up.

## Background

Follicular thyroid carcinoma (FTC) accounts for 10–20 % of differentiated thyroid carcinomas (DTCs), and it is the second malignant tumor originating from the follicular cells of the thyroid [1, 2]. In iodine-deficient areas, the relative rate of FTC tends to be higher, up to 40 % of all cases of DTC [3].

According to the World Health Organization (WHO) classification of thyroid tumors, FTC is defined by the presence of capsular and/or vascular invasion and by the absence of nuclear features typical of papillary thyroid carcinoma (PTC) [4]. FTC is more likely to metastasize to distant organs rather than to regional lymph nodes because of its tendency to invade blood vessels thus resulting in hematogenous dissemination [5].

WHO classification also divides FTC into minimally invasive follicular thyroid carcinoma (MI-FTC) when limited capsular and/or vascular invasion is found and widely invasive follicular thyroid carcinoma (WI-FTC) in the case of widespread infiltration of thyroid tissue and/or vascular invasion [4]. Several authors usually reported a benign clinical course of MI-FTC owing to the low risk of tumor recurrence and distant dissemination [1, 6–10]. Conversely, other authors described that also MI-FTCs can give rise to distant metastases [11].

WI-FTC is deemed to have a worse prognosis than MI-FTC [12, 13]. The subset of prognostic factors associated with WI-FTC, however, has not been deeply investigated yet [2].

Moreover, the impact of vascular invasion on prognosis is still a matter to be debated as some authors further subdivided MI-FTCs into angioinvasive and non-angioinvasive tumors [11, 14].

Age older than 45, sex, extrathyroid invasion, greater tumor size, and the presence of distant metastasis at presentation are recognized risk factors for poorer prognosis of FTC [5, 12, 15, 16]. Nevertheless, there is controversy concerning the prognostic significance of these risk factors, which could influence the treatment decision-making of patients with FTC [11].

As evidence on the biological behavior and clinical courses of MI-FTC and WI-FTC is still debatable, we carried out a retrospective cohort study in order to identify differences between MI and WI tumors and those prognostic parameters influencing late outcome such as recurrence and distant metastases. The current research was based on a single-institution experience, with homogeneity in the clinical management of patients with FTC.

## Methods

From January 1998 to October 2013, 556 consecutive patients were operated on in our surgical department because of a DTC. Among them, 71 (12.7 %) were patients with FTC and they represented the cohort of patients for the current study.

FTC was defined in accordance with the 2004 WHO classification of thyroid tumors as an invasive neoplasm of follicular cell origin without the typical nuclear features of PTC. Tumors were divided into MI-FTC when limited capsular and/or vascular invasion was found and WI-FTC in the case of widespread infiltration of thyroid tissue and/or blood vessels [4]. Tumors with ≤3 foci of vascular invasion were classified as MI-FTCs, whilst tumors with >3 foci of vascular invasion were classified as WI-FTCs. Hürthle cell thyroid tumors were excluded from the study as we consider oncocytic cell tumor as a distinctive clinical entity [17]. The follicular variant of papillary thyroid cancer was also excluded.

All patients underwent ultrasonography of both thyroid and neck before operation. Those patients with suspect of lymph node involvement by the tumor at ultrasonography underwent magnetic resonance imaging (MRI) of the neck and mediastinum.

Fine-needle aspiration cytology (FNAC) samples of the thyroid nodules were obtained under ultrasonographic guidance. Thyroid nodules showing cytological microfollicular features with severe atypia were considered as highly suspicious of FTC. The diagnostic categories of cytology were those of the British Thyroid Association (Thy 1, non-diagnostic; Thy 2, benign, non-neoplastic; Thy 3, follicular or suspected follicular or Hürthle neoplasm; Thy 4, suspicious of malignancy; Thy 5, diagnostic of malignancy) [18]. Only a few patients underwent immunocytochemical characterization of follicular nodules by testing the expression of galectin-3 and HBME-1 in FNAC samples. Lateral lymph node involvement was preoperatively assessed by means of both FNAC and/or cytological detection of thyroglobulin in cervical lymph nodes.

Intraoperative frozen section, when executed, was carried out with at least four sections obtained from the tumor. Thin anatomical slices (at least ten) were made to carefully analyze thyroid nodule. Metastatic lymph nodes were routinely examined with four histological sections.

We carried out a retrospective cohort study in order to compare 42 MI-FTC patients and 29 WI-FTC patients. The allocation of patients to the group MI or WI was based on the pathological epicrisis. The current study was conducted in accordance with the recommendations from the Strengthening the Reporting of Observational Studies in Epidemiology (STROBE) guidelines for reporting observational studies [19].

All the medical records were retrospectively reviewed. The comparison involved evaluation of patient characteristics (age and gender), tumor characteristics such as preoperative suspect of malignancy, tumor size, pathologic association, multifocality, thyroid capsular invasion, vascular invasion, extrathyroid invasion, lymph node involvement at presentation, the presence of a well/poorly differentiated tumor, tumor staging, and risk assessment. Pathologic cancer staging was in accordance with the pTNM (pathologic tumor node metastasis) system. Tumor risk was assessed according to the most common risk group system such as AMES (age, metastasis to distant sites, extrathyroidal invasion and size) [20] and AJCC (American Joint Committee on Cancer) pTNM staging following the 2010 pTNM-AJCC system [15].

Indications for surgical treatment, management characteristics, surgical complications such as inferior laryngeal nerve palsy and postoperative hypocalcemia, and follow-up results were also evaluated. Postoperative hypocalcemia was defined as a serum calcium level less than 8.0 mg/dl; patients were considered as having permanent hypoparathyroidism and permanent inferior laryngeal nerve palsy if the impaired function persisted after 6 months from the operation [21].

After discharge, all patients entered a scheduled clinical and instrumental follow-up program in the endocrinological department on an average duration of 113.0 months (range 12–288 months) and 125 months (range 24–196 months) in the MI and WI groups, respectively.

All patients diagnosed with a FTC underwent iodine-131 whole-body scan (WBS) 4–6 weeks after surgery, without L-thyroxin treatment and when the TSH level was higher than 30 μU/ml. A radioactive-iodine (RAI) ablating dose of 100 mCi (3700 MBq) was used to ablate microscopic thyroid remnants and micrometastases. A post-ablating WBS was done to search for missed metastases on the previous WBS. L-thyroxin suppressive treatment was started to maintain a serum TSH concentration of 0.1 μU/ml or less.

More recently, thyroglobulin (Tg) testing with human recombinant thyrotropin (rhTSH-Thyrogen®) was evaluated in a subgroup of patients prior to radioactive remnant ablation. Patients received 0.9 mg by intramuscular injection on days 1 and 2. Five mCi of iodine-131 were administered orally on day 3, and a WBS was performed on day 5. Determination of serum FT3, FT4, Tg, anti-thyroglobulin antibodies, and TSH were performed on day 1, prior to the first rhTSH injection. Tg and TSH levels were reassessed on days 3, 4, and 5.

Patients were followed-up at 6 and 12 months after treatment and then on a yearly basis. Only patients with tumor recurrence were resubmitted to RAI ablation therapy. Patients with undetectable TSH-stimulated serum Tg levels, without ultrasonographic evidence of neck lymph node metastases and negative WBS, were defined as cancer-free. Finally, 10-year overall and 10-year disease-free survivals were evaluated in both groups. Written informed consent was obtained from each patient to use data for scientific purposes. The current research was approved and supported by the review board of our Institution and it was conducted in accordance with the Declaration of Helsinki.

### Statistical analysis and synthesis of the results

Data were collected in a planned relational computer database (Microsoft Access®) including patients and tumor characteristics. All statistical analyses were carried out using the MedCalc® statistical software version 12.7.5 (MedCalc Software bvba, Ostend, Belgium; http://www.medcalc.org; 2013).

Data for tumor size and postoperative hospital stay were presented as the mean ± standard deviation (SD), as the median value and 95 % confidence interval (CI). Data were compared for statistical analysis using the Fisher exact test to evaluate differences between qualitative variables and using the Student’s *t*-test to compare quantitative variables. The objective of statistical analysis was also to identify independent risk factors significantly related to the presence of a WI-FTC, to the recurrence of tumors, and to the disease-free survival at 10-year follow-up by means of stepwise logistic regression analysis. Differences were considered significant when *p* < 0.05. *P* values of the study have been reported as calculated by the statistical software.

## Results

FTCs represented 12.7 % of all treated DTCs (71/556) during the whole period of the study. MI-FTC group included 36 women and 6 men (at a ratio of 6.0:1.0) with a mean age of 45.74 years (range 20–78 years). WI-FTC group consisted of 26 women and 3 men (at a ratio of 8.6:1.0) with a mean age of 48.48 years (range 19–75 years). Age and gender were not significantly different between groups. Moreover, the distribution of patients over and under 45 was similar in both groups.

The comparative cohort study revealed that there was no statistically significant difference regarding preoperative cytological diagnosis between groups. As shown in Table 1, 30 patients (71.4 %) with MI-FC and 15 patients (51.7 %) with WI-FTC were preoperative suspected as malignant by cytology and for this reason, total thyroidectomy was performed as a scheduled procedure. Altogether, the follicular neoplasm of 26 patients was characterized by immunocytochemical expression of galectin-3 and HBME-1 on FNAC sample, without significant difference between MI-FTC and WI-FTC (data not shown in tables). Five patients of the WI-FTC group were diagnosed with cervical lymph node metastasis. In this subgroup, cytological diagnosis of cervical lymph nodes was made in four patients, whilst high levels of lymph node thyroglobulin were detected in one patient (data not shown in tables).

**Table 1:** Tumor characteristics

Characteristics	MI-FTC	WI-FTC	*p*
N°	42	29	–
Suspect of malignancy by cytology (pre-op)	30 (71.4 %)^a^	15 (51.7 %)^b^	0.133
Tumor size (mm), mean ± SD	27.05 ± 11.52	39.34 ± 15.44	0.000
Range	6–52	16–75	
Median (95 % CI)	26 (23.57–30.53)	38 (33.72–44.96)	
Tumor size ≤ 1.0 cm	4 (9.5 %)	0	0.140
Tumor size > 1.0 cm ≤ 2.0	9 (21.4 %)	2 (6.8 %)	0.180
Tumor size > 2.0 cm ≤ 4.0	25 (59.6 %)	15 (51.8 %)	0.628
Tumor size > 4 cm	4 (9.5 %)	12 (41.4 %)	0.003
Pathologic association			
Multinodular goiter	13 (30.9 %)	10 (34.6 %)	0.800
Hashimoto’s thyroiditis	13 (30.9 %)	5 (17.2 %)	0.269
Graves’ disease	1 (2.4 %)	0	0.851
Normal thyroid	15 (35.8 %)	14 (48.2 %)	0.332
Follicular thyroid adenoma	1 (2.4 %)	0	0.851
Papillary microcarcinoma	6 (14.3 %)	3 (10.3 %)	0.729
Multifocality	0	2 (6.8 %)	0.163
Thyroid capsular invasion	0	1 (3.4 %)	0.408
Vascular invasion	9 (21.4 %)	15 (51.8 %)	0.011
Extra thyroid invasion	0	1 (3.4 %)	0.408
Lymph node metastasis	0	5 (17.2 %)	0.009
Well/Poorly differentiated	0	2 (6.8 %)	0.163
M1 at diagnosis	0	0	–
Concomitant parathyroid cancer	1 (2.4 %)	0	0.851

*MI-FTC* minimally invasive - follicular thyroid carcinoma, *WI-FTC* widely invasive - follicular thyroid carcinoma, *SD* standard deviation, *CI* confidence interval, *M1* metastasis to distant sites

^a^MI-FTC: 28 Thy 3, 2 Thy 4

^b^WI-FTC: 13 Thy 3, 2 Thy 4

Mean tumor size was significantly greater in the WI-FTC group than in the MI-FTC group (39.34 vs. 27.05 mm, *p* = 0.000). Indeed, WI-FTCs were significantly found with a diameter greater than 4.0 cm (*p* = 0.003) when compared to MI-FTCs. There was no statistically significant difference regarding pathologic thyroid association. At univariate analysis, vascular invasion and lymph node involvement at presentation were significantly associated to the presence of a WI-FTC (*p* = 0.011 and *p* = 0.009, respectively) (Table 1).

Higher cancer stage (III–IVA) was significantly related to the presence of a WI-FTC (*p* = 0.006). High risk of the AMES classification was significantly found in the WI-FTC group (*p* = 0.023) (Table 2). Patients of both groups were M0 at presentation.

**Table 2:** Tumor staging and risk assessment

Classification	MI-FTC	WI-FTC	*p*
N°	42	29	–
pTNM - AJCC Staging 2010			
I	23 (54.8 %)	11 (37.9 %)	0.024
II	13 (30.9 %)	4 (13.8 %)	0.156
III	6 (14.3 %)	13 (44.9 %)	0.006
IVA	0	1 (3.4 %)	0.408
AMES			
Low risk	32 (76.2 %)	14 (48.2 %)	
High risk	10 (23.8 %)	15 (51.8 %)	0.023

*MI-FTC* minimally invasive - follicular thyroid carcinoma, *WI-FTC* widely invasive - follicular thyroid carcinoma

Operative results, postoperative morbidity, and data about postoperative ablation therapy with iodine-131 have been reported in details in Table 3. Five patients of the WI-FTC group underwent total thyroidectomy associated with neck lymphadenectomy, whilst no patients of the MI-FTC group had more than total thyroidectomy. There was no difference between groups about postoperative hypocalcemia and postoperative laryngeal nerve palsy. None of the patients of this cohort had permanent hypoparathyroidism, and one patient of the WI-FTC group had permanent inferior laryngeal nerve palsy.

**Table 3:** Operative technique and postoperative results

Parameter	MI-FTC	WI-FTC	*p*
N°	42	29	
TT	40 (95.2 %)	21 (72.6 %)	
Completion thyroidectomy	2 (4.8 %)	3 (10.4 %)	
TT + CND	–	2 (6.8 %)	
TT + CND + MRND ipsilateral	–	1 (3.4 %)	
TT + CND + MRND bilateral	–	2 (6.8 %)	
Frozen section (true positive)	2 (4.8 %)	0	
Frozen section (false negative)	2 (4.8 %)	1 (3.4 %)	
Mean postoperative hospital stay			
(days) ± SD	3.7 ± 2.0	4.1 ± 2.5	0.520
Median (95 % CI)	3 (3.1–4.3)	3 (3.1–5.0)	
Morbidity			
Transient hypocalcaemia	6 (14.2 %)	4 (13.8 %)	0.773
Permanent hypocalcemia	–	–	
Transient unilateral laryngeal nerve palsy	–	–	
Permanent laryngeal nerve palsy	–	1 (3.4 %)	0.851
I131 ablation therapy			
0 application	–	–	–
1 application	37 (88.1 %)	22 (76.0 %)	0.209
2 applications	5 (11.9 %)	4 (13.8 %)	0.898
4 applications	–	1 (3.4 %)	0.064
5 applications	–	1 (3.4 %)	
8 applications^a^	–	1 (3.4 %)	
Tumor recurrence (node)	–	3 (10.4 %)	0.064
Lateral neck compartment	–	3 (10.4 %)	
Central neck compartment	–	–	
Loco-regional	–	1 (3.4 %)	
M1 at follow-up	–	3 (10.4 %)	0.064
Overall recurrence rate	–	7 (24.1 %)	0.001
Death of thyroid cancer	–	–	
Death of other causes	–	–	
Mean follow-up duration (months) (range)	113 (12–288)	125 (24–196)	

*MI-FTC* minimally invasive - follicular thyroid carcinoma, *WI-FTC* widely invasive - follicular thyroid carcinoma, *CND* central neck dissection, *MRND* modified radical neck dissection, *SD* standard deviation, *CI* confidence interval, *M1* metastasis to distant site

^a^Bone-marrow graft following aplastic anemia as side effect of multiple doses of RAI ablation therapy

After surgical treatment, all MI-FTC patients were submitted to a standard dose of 100 mCi of RAI ablation therapy. During follow-up, an additional dose of RAI ablation therapy was administered in five patients because of high thyroglobulin levels (>10 ng/ml) without evidence of tumor recurrence. All WI-FTC patients were submitted to a standard dose of 100 mCi of RAI ablation therapy. During follow-up, an additional dose of RAI ablation therapy was administered in one patient because of high thyroglobulin levels without evidence of tumor recurrence; three patients had an additional dose of RAI ablation after removal of nodal recurrence in the lateral neck compartment (15, 24, and 25 months after the operation, respectively); two patients had distant metastasis at 12 and 36 months after surgery, and they underwent a total of four and five doses of RAI ablation therapy, respectively. Finally, one patient with bilateral lymph node involvement had nodal and loco-regional recurrence 15 months after surgery and she was once more submitted to a dose of 100 mCi of RAI ablation therapy. Afterwards, she underwent a total of eight doses of RAI ablation therapy for the occurrence of distant metastases. At 156 months from the first operation, she is alive but she is not disease-free. Moreover, she was submitted to a bone-marrow graft following an aplastic anemia as side effect of multiple doses of RAI ablation therapy.

When analyzing seven patients with recurrence, three of them underwent total thyroidectomy and neck lymphadenectomy at first operation. In these patients, recurrence was found in cervical nodes, which were surgically removed before the second RAI ablation cycle. One more patient, who previously underwent total thyroidectomy alone, had a cancer recurrence in the thyroid bed and he was treated by only RAI ablation. Finally, three patients with distant metastases were treated by multiple doses of RAI ablation.

After a mean follow-up of 103.0 and 125.0 months in the MI and WI group, respectively, none of the patients in either group died because of FTC. Patients of the MI-FTC group had 100 % overall and disease-free survival at 10 years. Patients of the WI-FTC group had 100 % overall survival and 75.8 % disease-free survival at 10 years, respectively.

Multivariate analysis showed that a diameter >4 cm, the presence of vascular invasion, the TNM stage III–IVA and the high risk of AMES system risk stratification were independent prognostic factors significantly related to the presence of a WI-FTC (Table 4).

**Table 4:** Prognostic factors significantly related to the presence of a widely invasive FTC

Parameter	β	SE	*χ* ^2^	*p*	OR	95 % CI
Diameter >4 cm	1.758	0.650	8.306	0.006	5.805	1.632-20.764
Vascular invasion	1.368	0.528	7.027	0.009	3.928	1.393-11.072
Stage III–IVA	1.722	0.576	9.807	0.002	5.600	1.808-17.340
AMES High Risk	1.232	0.519	5.848	0.017	3.428	1.239-9.481

Stepwise logistic regression analysis

*β* coefficient, *SE* standard error, *χ* ^2^ chi square, *OR* odds ratio, *CI* confidence interval, *FTC* follicular thyroid carcinoma

Tumor size larger than 4.0 cm was the only factor retained in the multivariate statistical model as independent predictor of overall recurrence and influencing disease-free survival (Table 5).

**Table 5:** Independent risk factors for overall recurrence and disease-free survival after stepwise logistic regression analysis

Parameter	β	SE	*χ* ^2^	*p*	OR	95 % CI
Diameter >4 cm	1.909	0.967	3.903	0.048	6.750	1.01–44.92

*β* coefficient, *SE* standard error, *χ* ^2^ chi square, *OR* odds ratio, *CI* confidence interval

## Discussion

Pathological diagnosis, treatment policy, and prognosis of both MI-FTC and WI-FTC are still matters to be debated. Recently, the review of the European Society of Endocrine Surgeons has been published in an effort to reach an evidence-based consensus on the management of MI-FTC [22], whilst Ito et al. reported the clinical significance of specific prognostic factors of WI-FTC [2]. Within this regard, the current study was carried out in order to identify differences in clinical relevance between MI and WI tumors.

The main results of the current investigation showed that a diameter greater than 4.0 cm, the presence of vascular invasion, the TNM stage III–IVA, and the high risk at AMES system risk stratification were independent factors significantly related to the presence of a WI-FTC. The only factor retained in the multivariate statistical model as independent predictor of overall recurrence and disease-free survival at 10-year follow-up was a tumor size greater than 4.0 cm. Moreover, MI-FTC group showed a median diameter of 2.6 cm vs. 3.8 cm of the WI one and no patients of the MI group presented with tumor recurrence at follow-up.

Our data confirm other studies in which WI-FTCs larger than 4.0 cm (T3 on TNM staging system) have a worse prognosis because this diameter cut-off significantly affects cancer recurrence and cancer-specific survival [1, 2, 23]. In the case of greater tumors with a more likely aggressive biological behavior, a full treatment protocol including total thyroidectomy, RAI ablation, and L-thyroxin TSH-suppressive therapy and careful follow-up are strongly recommended [2, 16, 22].

Multivariate analysis showed vascular invasion as independently related to the presence of a WI-FTC. Indeed, the presence of extensive vascular invasion has been reported as associated with a higher risk of relapse in WI-FTCs [22]. Some authors highlighted the importance of vascular invasion in evaluating tumor prognosis by a sub-classification of FTCs. D’Avanzo et al. identified a group of MI-FTCs without vascular invasion (with 98 % of disease-free survival at 10 years), an intermediate MI-FTC group with vascular invasion and with or without capsular invasion (with 80 % of disease-free survival at 10 years), and a WI-FTC group (with 38 % of disease-free survival at 10 years) [13]. Similarly, O’Neil et al. showed that patients with MI-FTC without vascular invasion had a better disease-free survival at 40 months when compared with patients who had MI-FTC with vascular invasion or WI-FTC (97, 81, and 46 %, respectively) [6]. Several studies suggested that MI-FTC patients without vascular invasion could be treated by a more conservative approach in term of thyroid lobectomy [6, 7, 22, 24]. The choice of a less aggressive treatment could also be justified by the fact that the overall survival of patients with MI-FTC is essentially identical to that of the general population [25].

Conversely, other studies reported the occurrence of distant metastases also in MI-FTC patients without vascular invasion [6, 11, 26]. This means that MI-FTC could be a clinical dilemma being the most controversial issue to deal with. For these reasons, some authors considered the currently accepted histological classification of FTC as inadequate in predicting aggressive clinical courses [11]. The unclear and sometime unpredictable behaviors of MI-FTC persuaded these authors to recommend total thyroidectomy and RAI ablation for all patients with FTC [3, 11]. Also in our experience, all patients with MI-FTC were submitted to a full treatment protocol without relapse at 10 year follow-up.

According to other authors, we applied a more aggressive approach in all cases of FTC in terms of total thyroidectomy and RAI ablation therapy to facilitate the oncologic follow-up as well as the detection of occult metastases [3, 6, 11]. Our greatest concern was the possibility of overtreatment of patients belonging to the MI-FTC group. However, in the absence of molecular markers routinely used in predicting different biological behavior of FTC, which is finally diagnosed postoperatively, changing our treatment approach may be difficult.

Moreover, frozen section examination of FTCs has been proposed with the aim of modulating the treatment policy of these neoplasms. Some authors supported the use of frozen section in the management of FTCs because the diagnostic accuracy of malignancy was as high as 78 % [27], whilst several authors did not find any additional diagnostic value when using frozen section in the management of FTCs [28, 29]. In our experience, we performed only five intraoperative frozen sections because three of them were diagnosed as false negative requiring completion thyroidectomy.

Among several prognostic scoring systems, TNM staging is the most commonly used and it is considered as a good system for evaluating FTC [9, 26, 30]. However, the degree of invasiveness such as minimally invasive or widely invasive tumor, which is the worldwide-accepted classification of FTC, is not included in the TNM staging system [10]. Further studies are needed to better assess the impact of this discrepancy in the risk classification of FTC. Moreover, the updated TNM staging system has placed greater emphasis on the metastasis to lymph nodes in predicting prognosis of DTC [15]. In the case of FTC, there is a general agreement that nodal dissection must be performed only with therapeutic intent and in the presence of clinical evidence for lymph node involvement by the tumor [3, 17, 22]. In our practice, we plan neck lymph node dissection only after a preoperative detection of nodal involvement by the tumor (by nodal cytology or high nodal level of Tg). Indeed, when no nodal metastases can be detected preoperatively, there is no indication for prophylactic neck dissection in FTCs [22, 31–33]. Moreover, a significant correlation between tumor sizes of FTC greater than 4.0 cm and the presence of nodal metastases at presentation was demonstrated in previous studies [16, 33].

## Conclusions

The greatest clinical relevance of WI-FTC has been clearly showed by reviewing the literature and our own experience. Within this regard, more attention must be paid in the postoperative tumor re-staging of those patients with tumor size larger than 4.0 cm, which was the only parameter predicting overall recurrence and influencing disease-free survival. However, the unpredictable behaviors of MI-FTC and the need to facilitate the oncologic follow-up persuaded us to recommend a full treatment protocol for all patients with FTC.

An alternative, less aggressive operative strategy, performing intraoperative frozen sections, did not achieve any validation in our practice. Lymphadenectomy in FTCs should be performed only after a preoperative detection of nodal involvement by the tumor.

The good outcomes reported in the current study may be related to our quite aggressive therapeutic strategy. Nevertheless, definitive recommendations cannot be made without a longer follow-up. Finally, the inclusion of the degree of tumor invasiveness in the TNM staging system must be taken into account.

## Abbreviations

AMES: age, metastasis to distant sites, extrathyroidal invasion and size
DTC: differentiated thyroid cancer
FNAC: fine-needle aspiration cytology
FTC: follicular thyroid cancer
HBME-1: Hector Battifora mesothelial cell-1
MI-FTC: minimally invasive - follicular thyroid cancer
MRI: magnetic resonance
RAI: radioactive Iodine
Tg: thyroglobulin
TSH: Thyroid-stimulating hormone
WBS: whole body scintigraphy
WI-FTC: widely invasive - follicular thyroid cancer
